# A Narrative Review of Sex and Gender Differences in Sleep Disordered Breathing: Gaps and Opportunities

**DOI:** 10.3390/life12122003

**Published:** 2022-12-01

**Authors:** Margaret Bublitz, Nour Adra, Leen Hijazi, Fidaa Shaib, Hrayr Attarian, Ghada Bourjeily

**Affiliations:** 1 Department of Medicine, Warren Alpert School of Medicine, Brown University, Providence, RI 02903, USA; 2 Department of Psychiatry and Human Behavior, Warren Alpert School of Medicine, Brown University, Providence, RI 02903, USA; 3 Women’s Medicine Collaborative, The Miriam Hospital, Providence, RI 02906, USA; 4 Faculty of Medicine, American University of Beirut, Beirut 1107 2020, Lebanon; 5 Department of Medicine, Baylor College of Medicine, Houston, TX 77030, USA; 6 Department of Neurology, Northwestern University Feinberg School of Medicine, Chicago, IL 60611, USA

**Keywords:** sleep disordered breathing, obstructive sleep apnea, gender differences, sex characteristics

## Abstract

Introduction: Sleep disordered breathing (SDB) is a common condition, associated with multiple comorbidities including cardiovascular and metabolic disease. It has been previously established that SDB is more prevalent in men than women, shifting the literature’s focus away from the latter population. As such, underdiagnosis, and thus undertreatment, of SDB in women exists. Methods: To establish the differences in prevalence, clinical presentation, and pathophysiology of SDB between the two sexes, a narrative review of the current literature was performed. Results: Rates of SDB are higher among men, likely driven by differences in symptom presentation between men and women, with women presenting with more “atypical” symptoms, and lack of sensitivity in SDB screening tools to detect SDB in women. In addition to the cardiovascular risks of SDB, women with SDB may have worse quality of life, higher prevalence of insomnia, and respiratory issues. Discussion: More research is needed to better define the unique pathophysiology and clinical presentation of SDB in women. In addition, an increased awareness among health care providers and the lay public of the SDB-specific sex and gender differences will serve to minimize disparities in identification and treatment of SDB in women.

## 1. Introduction

Sleep disordered breathing (SDB) is a spectrum of disorders characterized by increased resistance to airflow in the upper airway, snoring, reduction in airflow (hypopnea), and cessation of breathing (apnea). Causes of SDB are likely multifactorial and may include excess weight/obesity, anatomical features of the jaw and neck, and genetic predisposition [1] SDB is a prevalent chronic health condition that is associated with host of morbidities including hypertension, pulmonary embolism, coronary heart disease, and heart failure [2]. SDB is also associated with increased risk of mortality [3].

Prevalence of SDB differs between men and women. For example, obstructive sleep apnea (OSA) has a 3 to 1 prevalence ratio for men compared to women [4]. The under -recognition and hence underdiagnosis and undertreatment of women with SDB has been recognized since the beginning of the century [5]. In fact, it takes women longer to get diagnosed with SDB compared to men [5]. According to the Society of Women’s Health Research (SWHR), only 1 in 4 women who suffer from OSA are diagnosed as such [6]. Despite some significant increase in recognition and diagnosis of SDB over the past decade, women with SDB continue to be overlooked, and therefore, untreated [7]. There have been recent significant efforts by the SWHR [6] and the National Institutes of Health (NIH) [8] to better understand sleep in women, including SDB, and to recognize, identify, and explore opportunities to address the impact of sex (a biological variable) and gender (a social variable) on sleep. These endeavors, among others, highlight the importance of integrating sex and gender into our understanding of various health conditions, including SDB.

Our understanding of sex and gender-based differences in prevalence, presentation, and pathobiology of SDB is still in its early stages. As such, the aim of this paper is to provide a narrative review of the literature on known sex and gender-based differences in SDB and highlight knowledge gaps that provide opportunities for future research studies.

## 2. Materials and Methods

We searched gender differences, sleep disordered breathing, obstructive sleep apnea and sex characteristics in PubMed and Medline. From the resulting 263, after removing duplicates and manual screening, only 78 articles remained which are referenced here.

## 3. Gender Differences in Clinical Presentation

Prevalence rates of SDB differ by gender; men have higher rates of SDB than women in most cohorts [9,10,11], however, there is evidence of clinicians’ bias in SDB diagnosis given that the largest differences in rates of SDB between men and women are reported in clinic-based research compared to population and community-based cohorts [5,12,13]. Gender biases also play a large role in the assessment of women with SDB. The “textbook” symptomatology of SDB, including snoring and apneas, often reflects symptoms that commonly occur in men. For example, likely due to multiple social factors, women are less likely to report snoring compared to men [14]. However, even when women experience similar SDB symptoms as men, they may still be dismissed or their symptoms attributed to other conditions such as mental health disorders or chronic fatigue [15]. Women generally tend to present with “atypical” SDB symptoms, such as daytime fatigue, nightmares, morning headaches, and mood disturbances [13,16]. Furthermore, the clinical presentation of SDB differs by gender, and this renders the commonly used screening tools less sensitive in identifying women with SDB, especially those with mild disease [17]. When classification of SDB is based on symptomatology, women with the disease are more likely to be categorized in the excessively sleepy and disturbed sleep subtypes when compared to men [17].

**Figure 1:**
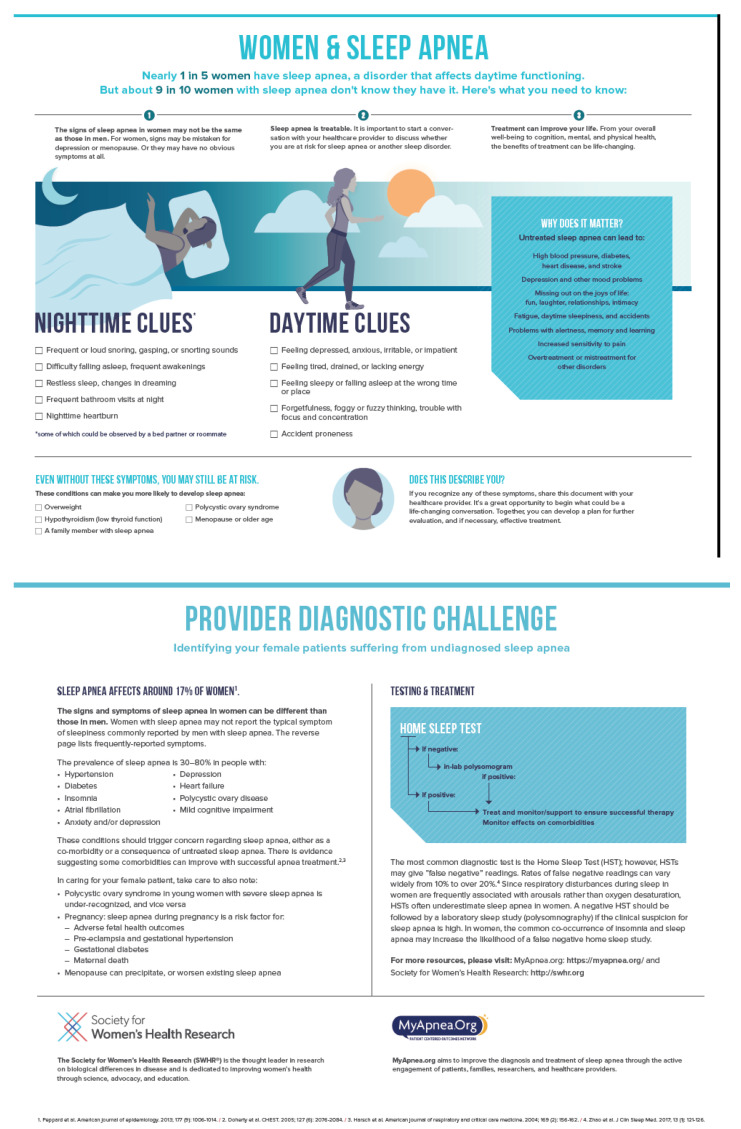
Used with permission from Society for Women’s Health Research. https://swhr.org/wp-content/uploads/2019/08/SWHR_Women-Sleep-Apnea_11.2017.pdf (accessed on 23 November 2022) [12,20,21,22].

Providers’ management of SDB has also been shown to differ by patients’ gender. As part of the Respiratory Health in Northern Europe (RHINE) study, nearly 11,000 men and women were asked about snoring, excessive daytime sleepiness, body mass index (BMI) and somatic diseases at baseline and at follow up 11 years later [18]. Among participants with symptoms of SDB, more men were diagnosed with OSA when compared to women (25% vs. 14%) [18]. In addition, female gender was associated with a lower probability of receiving treatment for SDB at follow up [18]. This provides additional evidence that SDB may be under-recognized, and hence, under-diagnosed and undertreated in women compared to men [19]. The SWHR has developed a two sided “cheat sheet” that is freely available on their website (https://swhr.org/swhr_resource/women-and-sleep-a-guide-for-better-health/ (accessed on 23 November 2022)) to educate both providers and patients on the presentation of SDB in women. See Figure 1. Although not yet validated, it can serve as a tool to increase awareness for both the clinician and the patient. Work is on the way to get this useful toolkit validated.

## 4. Gender Differences in Diagnostic Prediction of SDB Using Questionnaires

The most frequently used screening tools to identify patients with SDB include the Epworth Sleepiness Scale (ESS), the Snoring, Tired, Observed, Blood pressure, BMI, Age, Neck, Gender (STOP BANG) scale, and the Berlin Questionnaire (BQ). The performance of these screening tools may be different in men and women, both in the general population [16,23,24], and among specific clinical populations [25,26]. Data from the Sleep Heart Health study showed that, although women were as likely as men to report daytime sleepiness, women were less likely to have an abnormal score on the Epworth ESS [19]. More recent data from a Swedish cohort showed that only 34% of women with OSA have an abnormal ESS score [18]. A study examining the diagnostic performance of the STOP BANG and The Neck, Obesity, Snoring, Age and Sex (NoSAS) questionnaires concluded that the sensitivity of these two questionnaires in detecting sleep apnea in women was low [27]. Mou and colleagues (2018) recommended applying modified BMI and/or neck circumference thresholds using gender-triaged optimal operating points to improve specificity of the STOP BANG at detecting SDB [28]. In addition, the proportion of females in a population had a negative effect on the sensitivity of these questionnaires for mild, moderate and severe OSA indicating that scales are performing differently by gender [23]. In a sample of men and women matched on OSA severity, Pataka et al. (2020) reported that the BQ was highly sensitivity in detecting SDB in both men and women, and the STOP BANG and BQ equally predicted severe OSA across genders [29] (REF), suggesting that both gender and disease severity may impact performance of screening tools.

The sex or gender-based differences in the performance of screening tools, and particularly the reduced sensitivity of commonly used questionnaires, likely contribute to the difference in SDB prevalence observed between men and women [30]. Hence, the development or modification of questionnaires to reliably identify women at risk for having SDB is sorely needed to reduce bias in diagnosing this disorder in women. Figure 1 reproduced with permission is the SWHR toolkit.

## 5. Sex Differences in Anthropometrics, Phenotype, and Pathophysiologic Mechanisms

Four mechanisms have been described in the pathophysiology of SDB: upper airway (UA) anatomy, upper airway muscles’ ability to maintain an open airway, arousal threshold, and loop gain. The arousal threshold is the level of arousal needed to respond to a respiratory event. The loop gain depends on the stability of the respiratory system following a disturbance [31].

Furthermore, fluid shifts to the upper airway have been implicated in the pathogenesis of SDB [32]. Rostral fluid shifts during sleep correlate with changes in neck circumference, pharyngeal resistance, and upper airway patency, and therefore contribute to the pathogenesis of SDB [32]. Sex differences in fluid shifts have been observed. In a study of patients with heart failure, the change in neck circumference was smaller in women compared to men despite changes in leg fluids being similar between the two sexes [32]. Furthermore, in the same study, changes in leg fluid volume correlated inversely with changes in neck circumference and the apnea hypopnea index in men but not in women [32]. These differences suggest a different pathogenesis of upper airway patency in men and women. It is unclear whether these differences can be explained by sex hormones, given estrogen and progesterone’s role in fluid redistribution, as women in this study were post-menopausal.

The anatomical predisposition for SDB differ between men and women [33]. Women have a smaller number of alveoli, smaller lung volumes [34], a shorter diaphragm, as well as airways with smaller luminal areas when adjusted for height [35]. This constellation of physiological differences may explain why women have more SDB in the rapid eye movement (REM) phase of sleep, a state when the accessory respiratory muscles are paralyzed and breathing is solely diaphragm related [34]. Women have higher fat percentage than men, and the fat distribution is more peripheral [36]. Fat distribution varies with age in women [36]. After menopause, women’s fat distribution tends to become more central and visceral. Central and visceral adipose tissue is associated with both higher risk for SDB and a higher risk for developing cardiovascular disease [36].

Studies examining differences in ventilatory control and central and peripheral chemoreflex sensitivities show conflicting results among the two sexes [37]. Discrepancies can likely be attributed to differences in methodology, timing of testing in sleep versus wake state, and the lack of accounting for hormonal status. Through different pathophysiologic mechanisms, reproductive hormones play a role in the pathogenesis of SDB by impacting upper airway patency, contractility of the genioglossus muscle [38] and airway collapsibility, in addition to regulating fluid distribution [39,40]. Studies examining SDB in relation to the menstrual cycle showed that higher levels of progesterone are associated with lower upper airway resistance and respiratory disturbance index [39] suggesting a protective effect of this hormone. However, data on exogenous administration of estrogens and their role in the pathogenesis of SDB are conflicting due to differences in the products examined (natural versus synthetic, of animal or plant origin, estrogen alone or combined with progesterone, and dosing) [41,42]. It has also been hypothesized that any changes observed with exogenous hormonal replacement therapy may potentially reflect a pattern of healthy behavior rather than a direct hormonal effect [41,42]. On the other hand, testosterone increases the prevalence of, or worsens, SDB when administered to hypogonadal or older men [43,44]. In fact, polycystic ovary syndrome, a masculinizing condition in women, is associated with a significantly elevated risk of SDB [45,46], independent of weight [47]. Hence, there are many sex-differences in the pathogenesis and the potential phenotype of SDB that may have implications on therapy for SDB or for mitigating the risk of complications of SDB that need to be further examined.

## 6. Sex Differences in Polysomnographic Characteristics

**Figure 2:**
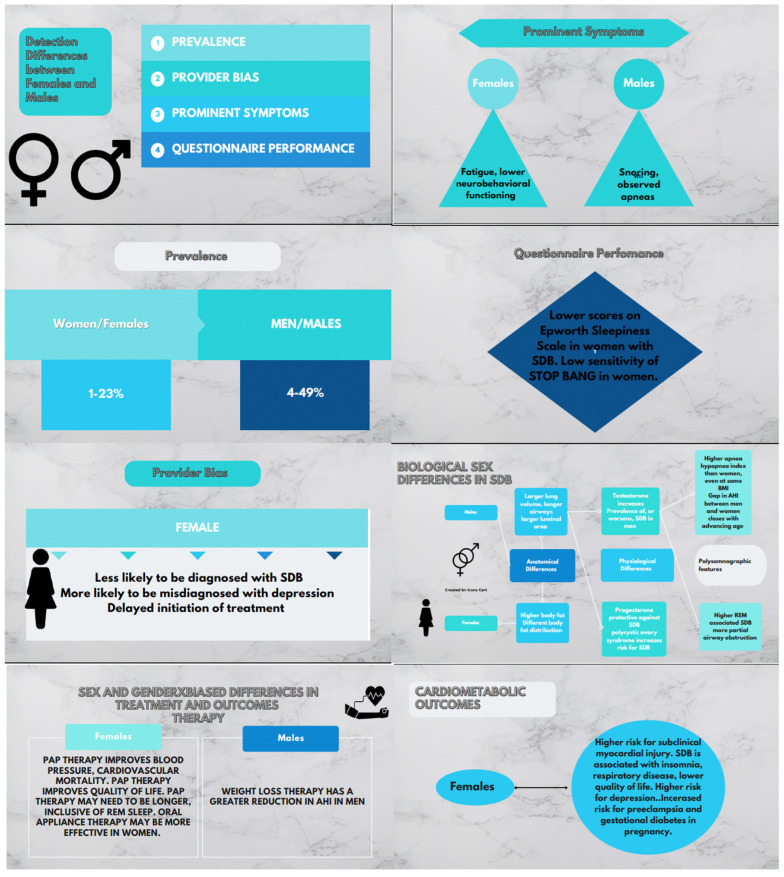
The role of sex and gender in sleep disordered breathing.

The anatomical and physiological sex differences translate into differences in polysomnographic parameters in SDB (Figure 2). Men have a higher apnea hypopnea index than women, even when controlling for BMI [48]. In fact, a recent study by Won et al. demonstrated a higher apnea-hypopnea index associated with ≥4% oxygen desaturation in men when compared to women [49]. However, the gap closes with advancing age [48,50,51]. On the other hand, some studies suggest that women have a higher prevalence of REM-related SDB compared to men [51,52]. A recent study by Mano et al. [53] studying a large Japanese population further demonstrated the higher incidence of OSA during REM sleep in women when compared to men. REM-related SDB has been linked to an increased risk of incident hypertension [54], metabolic syndrome, and diabetes [55]. Further, women have more partial airway obstruction than men and more airway obstruction that does not meet criteria for apnea or hypopnea [50].

## 7. Differences in the Association of SDB with Cardiovascular Outcomes

Data from two large cohorts show that, unlike men, women have a propensity for subclinical myocardial injury in response to SDB [56]. In fact, women with moderate to severe SDB have a higher risk of serious cardiovascular complications including heart failure and death, compared to those with mild or no SDB [57]. SDB in women may also interact with other risk factors of cardiovascular disease, such as smoking [58]. Furthermore, SDB is associated with insomnia, respiratory disease, and worse quality of life scores in women but not in men [59]. In addition, a recent study demonstrated that, after adjusting for the participants’ demographics and lifestyle factors, and despite women with SDB symptoms being often diagnosed with depression, male patients with moderate to severe OSA had higher risks of depression when compared to females [60].

Sex and reproductive hormones may influence the mechanisms linking SDB to cardiovascular outcomes in women. Estradiol plays an important role in the pathophysiology of cardiovascular outcomes in the setting of SDB. Animal studies have demonstrated that estrogens potentially downregulate oxidative stress pathways [61,62] and upregulate inflammatory pathways [63]. In addition, endothelial dysfunction may also be impacted by sex [64]. However, the influence of sex hormones on these pathways has not been specifically studied in humans as a potential mediator of adverse outcomes or as a therapeutic target.

## 8. SDB in Women during Specific Life Stages

It has been previously established that pregnancy is associated with a higher prevalence of SDB and that SDB is associated with pregnancy-specific outcomes such as preeclampsia and gestational diabetes [65,66] severe maternal morbidity [65,66] and adverse fetal and neonatal outcomes [67,68,69]. Though reproductive hormones may contribute to the pathobiology of SDB in pregnancy, other pregnancy-specific physical and biological factors may also play a role [70,71].

Furthermore, SDB prevalence is increased after menopause and is highly correlated with the increased incidence of insomnia in menopausal women [72]. A recent review reported that pre-menopausal and post-menopausal women on hormone replacement therapy (HRT), exhibited lower rates of OSA than post-menopausal women not receiving HRT [4]. Furthermore, the Nurse’s Health Studies compared naturally menopaused women to those surgically menopaused (through hysterectomy and oophorectomy) and demonstrated that the rate of OSA was higher in women who were surgically menopaused. This is most likely due to the abrupt decline in sex hormones with surgical menopause vs. natural menopause. The abrupt change does not allow for peripheral fat- based aromatization of androgens into estrogen [73]. Multiple reasons can explain the increase in SDB during this stage of a woman’s life. It has been well established that both estrogen and progesterone are two hormones that are involved in the regulation of breathing [17]. Progesterone, a prominent respiratory stimulant, declines as ovarian function deteriorates [61]. In addition, estrogens affect the functioning of the upper airway dilator muscles, and thus the drop in estrogen levels during menopause increases the risk of SDB [61]. The overall decline in these sex hormones also increase upper airway collapsibility. Furthermore, as women enter menopause, their fat redistributes to a male fat pattern, becoming more visceral, leading to an increase in the risk of developing SDB [41].

## 9. Treatment of SDB

The effect of sex and gender on the treatment of SDB has not been well studied. It has been shown that treatment of SDB is delayed in women compared to men. In fact, studies have demonstrated that women with SDB have a higher BMI than men at initiation of treatment. The literature on the impact of sex and gender on therapy outcomes is quite variable [56,74,75], however, a randomized controlled trial showed a positive effect of positive airway pressure (PAP) on blood pressure, but not on metabolic outcomes, in women [74]. One study compared an auto-titrating algorithm that was designed for women (higher sensitivity to airflow limitation and slower pressure rise in response to airflow limitation) to a standard auto-titrating algorithm [76]. The algorithm specifically designed for women demonstrated better control of airflow limitation. The mean hours of nightly PAP adherence, as well as prevalence of PAP compliance, are the same in both women and men [77]. Mean PAP requirements, however, are lower in women than men after controlling for severity of SDB and BMI [78]. As women appear to be more impacted by REM-related SDB than men and given the association of REM-related SDB with cardiovascular and metabolic outcomes, women may need to be treated for a longer nocturnal duration than men to ensure that PAP is applied during the latter part of the night when REM sleep is most prevalent.

Oral appliance therapy is an alternative to continuous PAP treatment in some patients. This therapy tends to be more effective in women than men, regardless of SDB severity [79]. However, the low proportion of women in many studies examining alternative to PAP therapy limits our ability to examine sex differences in treatment efficacy. The impact of weight loss on improvement in OSA demonstrates sex specific effects, with men having greater reduction in the apnea hypopnea index compared to women [80].

## 10. Conclusions

In summary, the current narrative review highlights disparities in the identification and treatment of SDB between men and women and provides targets for future work aimed at reducing SDB-associated morbidity across genders. It is currently unknown whether benefits of SDB treatment to reduce cardiovascular and metabolic outcomes very by sex and gender. Promising future directions include the development of modified screening tools that reflect the clinical picture of SDB through the different stages of women’s lives are needed. As well, sex-based modification of clinical protocols in which SDB therapy targets the latter part of the sleep cycle for women, where REM sleep is predominant, should be considered. Pathobiological facets of sex in SDB, as well as environmental and social aspects of gender, need to be better integrated into future research and training curricula [6,81]. An enhanced understanding of the effects of sex and gender could lead to the development of novel, personalized, sex-specific, mechanism-driven interventions to treat SDB.
